# Epidemiologic Profile of Hypertension in Northern Iranian Population: The PERSIAN Guilan Cohort Study (PGCS)

**DOI:** 10.5334/aogh.3027

**Published:** 2021-02-12

**Authors:** Mohammadreza Naghipour, Farahnaz Joukar, Arsalan Salari, Mehrnaz Asgharnezhad, Soheil Hassanipour, Fariborz Mansour-Ghanaei

**Affiliations:** 1 Gastrointestinal and Liver Diseases Research Center, Guilan University of Medical Sciences, Rasht, Iran; 2 Department of Cardiology, Cardiovascular Diseases Research Center, Heshmat Hospital, School of Medicine, Guilan University of Medical Sciences, Rasht, Iran; 3 Caspian Digestive Diseases Research Center, Guilan University of Medical Sciences, Rasht, Iran; 4 GI Cancer Screening and Prevention Research Center, Guilan University of Medical Sciences, Rasht, Iran

## Abstract

**Background:**

Estimates region-related prevalence of hypertension and attempts to identify its related factors at the district levels are required for prevention and management of hypertension.

**Objective:**

The aim of this study was to investigate the epidemic features and related factors of hypertension and its awareness, treatment, and control rates among the northern Iranian population.

**Methods:**

It was a community based cross-sectional study based on data from PERSIAN Guilan Cohort Study (PGCS). In total, 10,520 participants (aged 35–70 years) from the Guilan Province in northern Iran included in this study, between October 8, 2014, and January 20, 2017. Hypertension was defined as systolic blood pressure ≥140 mmHg or diastolic blood pressure ≥90 mmHg or a prior diagnosis of hypertension or being on antihypertensive medication. Potential correlates of hypertension and its awareness, treatment and control were analyzed by multivariate logistic regression adjusted for demographic factors, anthropometric characteristics, lifestyle variables, past medical history, and laboratory data.

**Results:**

The prevalence of hypertension was 43.2% and the hypertension awareness, treatment, and control rate were 53.4%, 49.8%, and 73.7%, respectively. The multivariate logistic regression analyses revealed that older age, urbanization, lower education, overweight and obesity, lower physical activity, prediabetes and diabetes, cardiovascular disease, psychiatric disorder, positive family history of hypertension and raised serum creatinine were independently associated with presence of hypertension. Awareness of hypertension was greater in the female sex, older age, rural residency, higher education and patient with comorbidities. Older age, rural residency and comorbidities were associated with treatment of hypertension. Control of hypertension was better among younger age, higher education, normal weight and higher physical activity.

**Conclusion:**

Hypertension is highly prevalent in the northern Iranian population. About half of affected persons are unaware of their disease and untreated. Modifying risk factors (such as weight lose and increase physical activity) and increasing hypertension awareness (by screening) is essential for primary and secondary prevention of high blood pressure in this population, especially in urban areas and among males, younger ages, and less educated.

## Introduction

Hypertension is one of the important public health concerns worldwide, due to its high prevalence and its association with cardiovascular disease [1,2]. Detection, control and monitoring of hypertension are major health challenges throughout the world [3,4,5]. The prevalence of hypertension is increasing constantly in developing countries over recent decades in line with the population aging process and rapid economic development [6,7,8]. Based on national STEPS (stepwise approach in surveillance of non-communicable diseases) survey in Iran, during 2016, 27.01 % of the Iranian adult population ≥25 years of age suffered from hypertension [9,10]. There is a meaningful difference in hypertension prevalence in various regions of Iran from 19% to 40% due to the impact of cultural and economic diversity and complex geographical patterns and It is more prevalent in northern Iran [9].

Hypertension is a multifactorial disease associated with increased age [11], low socioeconomic status [12], family history of hypertension [13], and behavioral risk factors including smoking, alcohol drinking, poor dietary habits, high body mass index (BMI) [14], sedentary lifestyle, poor stress management [15], unavailability of health care and basic medical treatment [16]. Some studies have shown that public health interventions and lifestyle modification among different populations have effects regarding the management of hypertension [17,18,19]. However, the precise role and effect value of these risk factors in the prediction of hypertension remain unclear.

Hence, estimates region-related prevalence of hypertension and attempt to identify its related factors at the district levels are required for policy making, intervention priorities setting, to local programs evaluating and to help formulate and devise approaches in the prevention and management of hypertension in various regions. Therefore, the current study was planned to investigate the epidemic features and related factors of hypertension and its awareness, treatment and control rates among the northern Iranian population. This study was based on data from The PERSIAN Guilan Cohort Study (PGCS), a prospective, population-based cohort study in Guilan, Iran.

## Methods

### Study Population and Study Design

It was a community based cross-sectional study based on data from The PERSIAN Guilan Cohort Study (PGCS) [20], which were conducted in Guilan, the northern province of Iran, recruited between October 8, 2014, and January 20, 2017, as part of the Prospective Epidemiological Research Studies in Iran (PERSIAN) [21]. The main goal of the of the PERSIAN study was: (a) to determine the prevalence and incidence of non-communicable diseases, (b) to compare the relationships between risk factors and NCDs, (c) to establish a Biobank for basic scientific research [22].

The full details of the study and validations have been described elsewhere [20,22,23,24]. In short, different districts of the Guilan province were chosen to include different socioeconomic status levels including urban areas and 39 villages. This area was selected due to its long-term population stability, high population density, a relative similarity in demographic and behavioral characteristics. In total, 10520 participants (aged 35–70 years) included in this study. The response rate was 83.2% [20]. The study was approved by the ethical committee of Guilan University of Medical Sciences (ethical code: IR.GUMS.REC.1397.371). Written informed consent was obtained from all participants prior to the enrolment in the studies and privacy and confidentiality of data and autonomy of participants were considered [20].

### Data Collection

Data was collected using a face-to-face interview format and a physical examination by trained interviewers [20]. Age, sex, educational level and habitat were collected as demographic variables. Information on Lifestyle habits, including Smoking status (nonsmoker vs current smoker of tobacco, cigarettes, hookah, cigar, or pipe), alcohol consumption (none or currently use), level of physical activity (metabolic equivalent tasks (METs) of self-reported daily activities) were collected [25]. Anthropometric characteristics, including weight (kg), Height (cm), waist circumferences (cm) are measured using US National Institutes of Health protocols and have been previously described in detail [20]. Body mass index (BMI) was considered as weight in kilograms/height in meters squared. Overweight and obesity were defined as BMI ≥ 25–29.9 kg/m^2^ and BMI ≥ 30 kg/m^2^, respectively [26]. Abdominal obesity was defined as waist circumferences ≥88 in both males and ≥ 102 in females [27]. Laboratory tests, including serum total cholesterol (TC), triglyceride (TG), low-density lipoprotein cholesterol (LDL-C), high-density lipoprotein cholesterol (HDL-C) and creatinine was measured. Raised total cholesterol was defined if serum TC ≥ 200 mg/dL [28]. Raised triglyceride was defined if serum TG ≥ 150 mg/dL [29]. Raised LDL-C was defined if serum LDL-C ≥ 130 mg/dL [28]. Past medical history consisted of diabetes mellitus, cardiovascular disease, psychiatric disorder diagnosed by a physician and self-reported were recorded.

### Blood Pressure Measurement

Blood pressure (mmHg) was measured after 10 min rest period twice in each arm supported at heart level with participants in a seated position with their back supported and legs uncrossed in a quiet room after ten-minute intervals, using Richter auscultator mercury sphygmomanometers (MTM Munich, Germany) with appropriately adjusted cuff size [22]. The average of the measurements was used in the analyses.

Hypertension was defined as systolic blood pressure ≥140 mmHg and/or diastolic blood pressure ≥90 mmHg and/or a prior diagnosis of hypertension by a health professional or being on antihypertensive medication [20]. Awareness of hypertension was regarded as the proportion of hypertensive participants who self-reported any prior diagnosis of hypertension by a health care professional. The proportion of hypertensive participants who were receiving prescribed antihypertensive drugs for high BP controlling was considered as treated. Controlled hypertension was defined as the proportion of hypertensive participants on antihypertensive medication with systolic blood pressure <140 mm Hg and diastolic blood pressure <90 mm Hg [30].

### Statistical Analysis

Differences in characteristics of the participants according to the presence of hypertension (normotensive, hypertensive), awareness, treatment, and control of hypertension were analyzed using the chi-square test, Fisher’s Exact Test for categorical variables. Normality of continuous variables was evaluated by Kolmogorov-smirnov test and independent Student’s t test was used for normal continuous variables. Not normally distributed data were analyzed using mann-whitney U test.

Multiple logistic regression models were used to determine independent correlated factors (independent variables) of presence, awareness, treatment, and control of hypertension (dependent variables) and adjusted odds ratios and 95% CI were calculated. All variables with a p value less than or equal to 0.2 in univariate analysis were examined in a multivariate logistic regression model. The data were analyzed using SPSS version 17.0 (SPSS Inc., Chicago, IL, USA). A P-value of less than 0.05 was considered as significant.

## Result

### Sample Characteristics

Of the total 10,520 subjects who participated in the study, 46.5% were men. The mean age of the study population was 51.51 ± 8.8 years and 43.8% of them were urban residents. Thirty–nine percent of the participants were educated high school or less.

**Figure 1:**
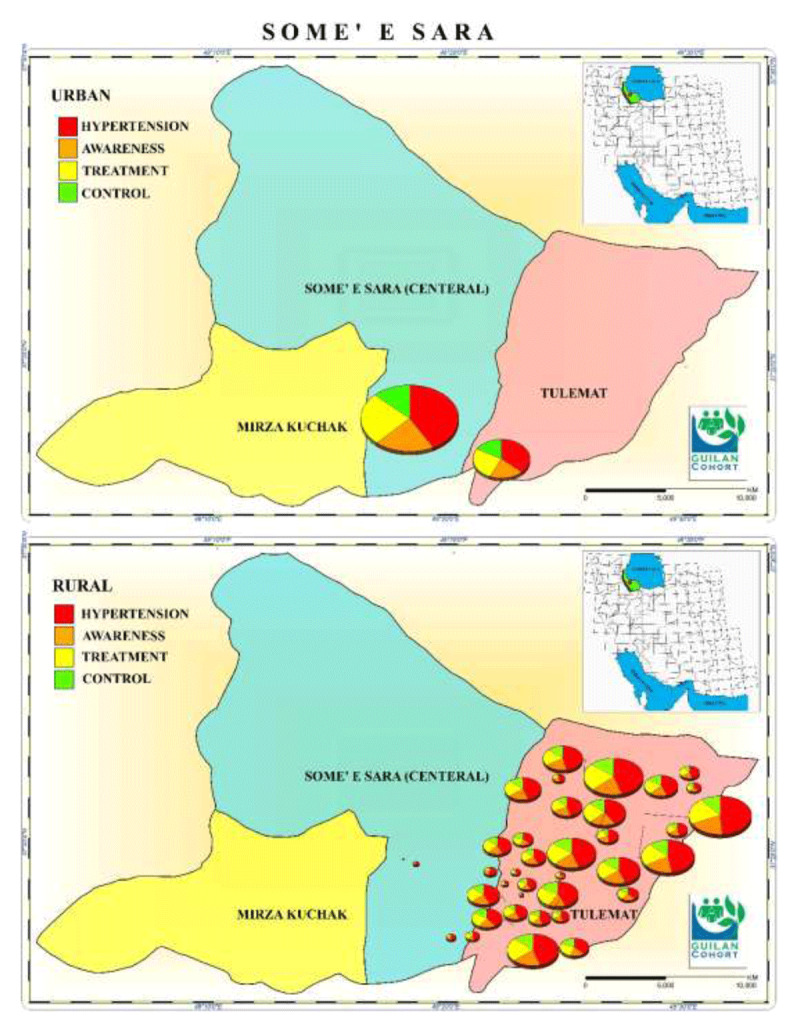
Geographic distribution of Hypertension prevalence, awareness, treatment and control in urban and rural area of Guilan cohort study. (The map depicted in figure is our own work). Data are expressed as mean ± standard deviation or number (percentages).

The mean systolic BP level of the study population was 118.25 ± 16.7and mean diastolic BP level was 77 ± 11. The prevalence of hypertension was 43.2% and the hypertension awareness, treatment, and control rate were 53.4%, 49.8%, and 73.7%, respectively (***Table 1***). Geographic distribution of hypertension prevalence, awareness, treatment and control in urban and rural area of Guilan cohort study is presented in ***Figure 1***.

**Table 1:** Characteristics of study population according to hypertension presence, awareness, treatment, and control.


VARIABLES	HYPERTENSION				AWARENESS				TREATMENT				CONTROL		
			
	NORMOTENSIVE (N = 5977)	HYPERTENSIVE (N = 4543)	P-VALUE*		UNAWARE (N = 2118	AWARE(N = 2425)	P-VALUE*		UNTREATED (N = 2281)	TREATED (N = 2262)	P-VALUE*		UNCONTROLLED (N = 593)	CONTROLLED (N = 1669)	P-VALUE*

Diastolic BP (mmHg)	74.3 ± 10	80.5 ± 11	<0.001		76.9 ± 10	83.6 ± 10	<0.001		81.1 ± 11	79.9 ± 10	<0.001		92.1 ± 7	75.5 ± 8	<0.001

Systolic BP (mmHg)	113.5 ± 14	124.8 ± 18	<0.001		116.8 ± 14	131.1 ± 17	<0.001		124.9 ± 18	124 ± 17	0.1		145.8 ± 15	116.3 ± 11	<0.001

Age (year)	49.5 ± 8	54.1 ± 9	<0.001		52.2 ± 8	55.6 ± 8	<0.001		53.4 ± 9	54.7 ± 8	<0.001		56.9 ± 8	53.9 ± 8	<0.001

Urban (%)	2427(40.6%)	2186(48.1%)	<0.001		1131(53.4%)	1055(43.5%)	<0.001		1213(53.2%)	973(43%)	<0.001		232(39.1%)	741(44.4%)	0.01

High school or less education level (%)	2108(35.3%)	1995(43.9%)	<0.001		1044(49.3 %)	917(37.8 %)	<0.001		1022(44.8%)	973(43%)	0.1		288(48.6%)	685(41%)	0.001

Male (%)	2983(49.9%)	1904(41.9%)	<0.001		1123(53%)	781(32.2%)	<0.001		938(41.1%)	966(42.7%)	0.1		261(44%)	705(42.2%)	0.2

BMI (kg/m^2^)	27.6 ± 5	28.7 ± 5	<0.001		27.5 ± 4	29.8 ± 5	<0.001		28.8 ± 5	28.6 ± 5	0.2		29.6 ± 5	28.3 ± 5	<0.001

Waist circumference (cm)	97.3 ± 12	100.7 ± 12	<0.001		96.8 ± 11	104 ± 1 ± 11	<0.001		100.5 ± 12	100.9 ± 12	0.2		103 ± 11	100 ± 11	<0.001

Physical activity(METs/hour/day)	42.2 ± 9	39.9 ± 8	<0.001		41.3 ± 8	38.6 ± 7	<0.001		40.1 ± 8	39.7 ± 8	0.09		38.6 ± 7	40.1 ± 8	<0.001

Use of Alcohol (%)	941(15.7%)	574(12.6%)	<0.001		336(15.9%)	238(9.8%)	<0.001		269(11.8%)	305(13.5%)	0.04		73(12.3%)	232(13.9%)	0.1

Smoking (%)	1498(25.1%)	1086(23.9%)	0.09		618(29.2%)	468(19.3%)	<0.001		525(23%)	561(24.8%)	0.8		136(22.9%)	425(25.5%)	0.1

Diabetic	936(15.7%)	1595(35.1%)	<0.001		601(28.4%)	994(41%)	<0.001		661(29%)	934(41.3%)	<0.001		270(45.5%)	664(39.8%)	0.01

Has cardiovascular disease	175(2.9%)	580(12.8%)	<0.001		163(7.7%)	417(17.2%)	<0.001		201(8.8%)	379(16.8%)	<0.001		97(16.4%)	282(16.9%)	0.4

Has Psychiatric disorder	715(12%)	914(20.1%)	<0.001		316(14.9%)	598(24.7%)	<0.001		443(19.4%)	471(20.8%)	0.1		126(21.2%)	345(20.7%)	0.4

Positive Family history of hypertension	3587(60%)	3317(73%)	<0.001		1412(66.7%)	1905(78.6%)	<0.001		1665(73%)	1652(73%)	0.5		122(20.6%)	488(29.2%)	<0.001

TC (mmol/L)	193.1 ± 38	192.3 ± 39	0.3		193 ± 38	191.3 ± 40	0.07		195.1 ± 39	189.6 ± 40	<0.001		192 ± 40	188.5 ± 40	0.02

TG (mmol/L)	155.3 ± 10	166.6 ± 10	<0.001		164.1 ± 10	168 ± 10	0.1		165.8 ± 10	167.4 ± 10	0.6		176.4 ± 11	164.2 ± 10	0.01

HDL-C (mmol/L)	48.5 ± 11	48.1 ± 10	0.1		48.5 ± 10	47.8 ± 10	0.06		48.4 ± 10	47.9 ± 10	0.1		47.7 ± 10	48 ± 10	0.6

LDL-C (mmol/L)	113.9 ± 30	111.3 ± 33	<0.001		112.6 ± 31	110.2 ± 34	0.01		113.8 ± 32	108 ± 33	<0.001		110 ± 34	108 ± 33	0.09

Serum creatinine (µmol/L)	0.89 ± 0.1	0.9 ± 0.1	<0.001		0.89 ± 0.1	0.9 ± 0.1	<0.001		0.9 ± 0.1	0.9 ± 0.1	0.3		0.91 ± 0.1	0.9 ± 0.1	0.08


* Statistical significance based on the independent Student’s t tests for continuous variables or Chi-square or Fisher’s Exact test for categorical variables. Abbreviations: BP: blood pressure; BMI: body mass index; METs: metabolic equivalent rates; TC: total cholesterol; TG: triglyceride; HDL-C: high-density lipoprotein cholesterol; LDL-C: low-density lipoprotein cholesterol. Definitions: Hypertension: systolic BP ≥ 140 mmHg and/or diastolic BP ≥ 90 mmHg and/or a prior diagnosis of hypertension by a health professional or being on antihypertensive medication; Awareness: hypertensive patient who self-reported any prior diagnosis of hypertension by a health care professional; Treatment: hypertensive patient who were receiving prescribed antihypertensive drugs for high BP controlling; control: hypertensive patient on antihypertensive medication with systolic BP < 140 mm Hg and diastolic BP < 90 mm.

#### Hypertension prevalence related factors

The characteristics of the study population, according to hypertension presence are presented in ***Table 1***. Hypertensive subjects were significantly more likely to be female, from urban areas, had a high school or less education level, and to be older age (all P value < 0.001) (***Table 1***). Also, the mean of systolic and diastolic BP, BMI, waist circumference were significantly higher in hypertensive vs normotensive participants (all P value < 0.001) (***Table 1***). Medical history of diabetes, cardiovascular disease, psychiatric disorder and family history of hypertension were significantly more common among hypertensive subjects (all P value < 0.001) (***Table 1***). In lifestyle aspects, physical activity and alcohol use were significantly lower in hypertensive participants and but the prevalence of smoking was not significantly different between two groups (***Table 1***). Comparison of laboratory parameters showed a significant increase in triglyceride, LDL and serum creatinine in the hypertensive participants vs normotensive (all P value < 0.001) (***Table 1***) but there was no significant difference in total cholesterol and HDL (***Table 1***).

***Table 2*** demonstrates the results multivariate logistic regression to explore the independent correlates of hypertension presence. The multivariate analyses revealed that older age, urbanization, lower education, over weight and obesity, lower physical activity, prediabetes and diabetes, cardiovascular disease, psychiatric disorder, positive family history of hypertension and raised serum creatinine were significantly independently associated with presence of hypertension (all P value < 0.001) (***Table 2***).

**Table 2:** Multivariable logistic regression analysis of variables associated with hypertension presence, awareness, treatment, and control.


VARIABLES	HYPERTENSION PRESENCE				AWARENESS				TREATMENT				CONTROL		
			
	AOR*	(95%CI)	P-VALUE		AOR*	(95%CI)	P-VALUE		AOR*	(95%CI)	P-VALUE		AOR*	(95%CI)	P-VALUE

Age(year)															

35–50(ref)	1				1				1				1		

51–70	2.1	1.9–2.3	<0.001		1.8	1.6–2.1	<0.001		1.2	1.1–1.3	0.002		0.6	0.5–0.8	<0.001

Residence															

Rural (ref)	1				1				1				1		

Urban	1.4	1.2–1.5	<0.001		0.7	0.6–0.8	<0.001		0.6	0.5–0.7	<0.001		1	0.8–1.2	0.6

Educational level															

high school or less(ref)	1				1				–				1		

diploma or more	0.8	0.7–0.9	<0.001		1.4	1.3–1.6	<0.001		–	–	–		1.4	1.2–1.8	<0.001

Sex															

Male(ref)	1				1				–				–		

Female	1	0.8–1.1	0.6		1.3	1.1–1.6	0.008		–	–	–		–	–	–

BMI															

Normal(ref)	1				1				–				1		

Overweight	1.2	1.1–1.3	0.001		1.4	1.2–1.7	<0.001		–	–	–		0.6	0.5–0.9	0.008

Obese	1.4	1.2–1.6	<0.001		1.8	1.4–2.2	<0.001		–	–	–		0.4	0.3–0.6	<0.001

Waist circumference															

normal(ref)	1				1				–				1		

Abdominal obesity	1	0.9–1.2	0.4		1.6	1.3–2	<0.001		–	–	–		0.8	0.5–1.1	0.2

Physical activity (METs/hour/day)															

<34.8(Q1)(ref)	1				1				–				1		

34.8–38.8(Q2)	0.9	0.8–1.1	0.5		0.7	0.6–0.9	0.006		–	–	–		1.2	0.9–1.5	0.1

38.8–45.8(Q3)	0.7	0.7–0.8	<0.001		0.6	0.5–0.7	<0.001		–	–	–		1.2	0.9–1.5	0.1

≥45.8(Q4)	0.6	0.5–0.6	<0.001		0.6	0.5–0.8	<0.001		–	–	–		1.6	1.2–2.2	0.001

Alcohol use															

No(ref)	1				1				1				–		

Yes	1	0.8–1.1	0.6		0.8	0.7–1.1	0.8		1.1	0.9–1.4	0.07		–	–	–

Smoking															

No(ref)	–				1				–				–		

Yes	–	–	–		0.9	0.8–1.1	0.8		–	–	–		–	–	–

Diabetes															

No	1								1				1		

Prediabetes	1.4	1.2–1.6	<0.001		0.9	0.8–1.1	0.6		1	0.8–1.2	0.4		1	0.7–1.3	0.8

Diabetic	2.1	1.9–2.3	<0.001		1.5	1.2–1.7	<0.001		1.6	1.4–1.9	<0.001		1.1	0.9–1.4	0.1

Cardiovascular disease															

No(ref)	1				1				1				–		

Yes	3.1	2.5–3.7	<0.001		2	1.6–2.4	<0.001		1.8	1.5–2.2	<0.001		–	–	–

Psychiatric disorder															

No(ref)	1				1				–				–		

Yes	1.5	1.3–1.6	<0.001		1.5	1.2–1.7	<0.001		–	–	–		–	–	–

Family history of hypertension															

No(ref)	1				1				–				1		

Yes	1.8	1.6–1.9	<0.001		1.8	1.6–2.1	<0.001		–	–	–		1.6	1.2–2	<0.001

TC (mmol/L)															

Normal	–				–				1				1		

Raised	–	–	–		–	–	–		0.9	0.8–1.2	0.9		0.9	0.8–1.1	0.07

TG (mmol/L)															

Normal	1				–				–						

Raised	1.1	0.9–1.2	0.06		–	–	–		–	–	–		1	0.8–1.1	0.8

LDL-C (mmol/L)															

Normal	1				1				1				–		

Raised	1	0.8–1.06	0.6		0.9	0.8–1.07	0.5		0.8	0.7–1.1	0.07		–	–	–

Serum creatinine (µmol/L)															

Normal	1				1				–				–		

Raised	5.1	2.1–12	<0.001		2	0.9–4.4	0.06		–	–	–		–	–	–


* Adjusted Odds ratio: Adjusted for all variables that were significant in univariate analyses. CI: confidence interval. Abbreviations: BP: blood pressure; BMI: body mass index; METs: metabolic equivalent rates; TC: total cholesterol; TG: triglyceride; HDL-C: high-density lipoprotein cholesterol; LDL-C: low-density lipoprotein cholesterol. Definitions: Hypertension: systolic BP ≥ 140 mmHg and/or diastolic BP ≥ 90 mmHg and/or a prior diagnosis of hypertension by a health professional or being on antihypertensive medication; Awareness: hypertensive patient who self-reported any prior diagnosis of hypertension by a health care professional; Treatment: hypertensive patient who were receiving prescribed antihypertensive drugs for high BP controlling; control: hypertensive patient on antihypertensive medication with systolic BP < 140 mm Hg and diastolic BP < 90 mm.

### Awareness of Hypertension Related Factors

The awareness rate in hypertensive participants was 53.4%. Subjects who were aware of their hypertension was significantly more likely to be female, from rural areas, had a high school or less education level, and to be older age (all P value < 0.001) (***Table 1***). Also, the mean of BMI, waist circumference were significantly higher in aware versus unaware participants (all P value < 0.001) (***Table 1***). Medical history of diabetes, cardiovascular disease, psychiatric disorder and family history of hypertension were significantly more common among aware subjects (all P value < 0.001) (***Table 1***). In lifestyle aspects, physical activity and alcohol use and smoking was significantly lower in aware participants (all P value < 0.001) (***Table 1***). Comparison of laboratory parameters showed a significant increase in serum creatinine and a significant decrease in LDL in the aware versus unaware participants (***Table 1***), but there was no significant difference in total cholesterol, triglyceride and HDL (***Table 1***).

The results multivariate logistic regression to explore the independent correlates of awareness of hypertension was shown in ***Table 2***. The multivariate analyses revealed that female sex, older age, rural residency, lower education, over weight and obesity, abdominal obesity, lower physical activity, diabetes, cardiovascular disease, psychiatric disorder, and positive family history of hypertension were significantly independently associated with awareness of hypertension (all P value < 0.05) (***Table 2***).

### Treatment of Hypertension Related Factors

The treatment rate in hypertensive participants was 49.8%. Subjects who was treated were significantly more likely to be from rural areas and older age (all P value < 0.05) (***Table 1***). Sex and education level were not associated with treatment of hypertension (***Table 1***). The mean of diastolic BP was significantly lower in treated patient (P value < 0.001) (***Table 1***) but mean of systolic BP, BMI, waist circumference were not significantly different between treated and untreated patient (***Table 1***). Medical history of diabetes, cardiovascular disease was significantly more common among treated subjects (all P value < 0.001) (***Table 1***). In lifestyle aspects, alcohol use was significantly higher in treated participants but physical activity and smoking were not significantly different between two groups (***Table 1***). Comparison of laboratory parameters showed a significant decrease in total cholesterol and LDL in the treated participants (all P value < 0.05) (***Table 1***), but there was no significant difference in triglyceride and HDL and serum creatinine (***Table 1***).

The results multivariate logistic regression to explore the independent correlates of treatment of hypertension was shown in ***Table 2***. The multivariate analyses revealed that older age, rural residency, diabetes and cardiovascular disease were significantly independently associated with treatment of hypertension (all P value < 0.05) (***Table 2***).

### Control of Hypertension Related Factors

The control rate in treated hypertensive participants was 73.3%. Subjects who treated more likely younger age, urban residency and higher educational level were significantly associated with control of hypertension (all P value < 0.05) but there were no significant associated with sex (***Table 1***). The mean of systolic and diastolic BP, BMI, Waist circumference were significantly lower in controlled versus uncontrolled patients (all P value < 0.001) (***Table 1***). Medical history of diabetes was significantly less common among and family history of hypertension were significantly more common among controlled subjects (all P value < 0.001) (***Table 1***). In lifestyle aspects, physical activity was significantly higher in controlled participants (P value < 0.001) but alcohol use and smoking were not significantly different between two groups (***Table 1***). Comparison of laboratory parameters showed a significant decrease in total cholesterol and triglyceride in the controlled participants (all P value < 0.05) (***Table 1***), but there was no significant difference in LDL and HDL and serum creatinine (***Table 1***).

The results multivariate logistic regression to explore the independent correlates of control of hypertension was shown in ***Table 2***. The multivariate analyses revealed that younger age, higher educational level, normal weight, physical activity more than 45.8 METs/hour/day and positive family history of hypertension were significantly independently associated with control of hypertension (all P value < 0.05) (***Table 2***).

## Discussion

The current study investigated the prevalence and related factors of hypertension and its awareness, treatment and control rates among the northern Iranian population. In this study, we demonstrate that hypertension was prevalent (43.2%) in this population. Although the prevalence of hypertension in the current study population was similar to other studies in north of Iran [31], but it was more prevalent than most of the other regions of Iran and other countries [32,33]. The recent studies reported that the prevalence of hypertension overall in Iran was about 25% [34,35], and its prevalence in southern province was 27% [36]. This difference may be related to differences in demographic and lifestyle factors [37].

About half of the hypertensive patients in the current study were aware of their disease and under treatment and about two-thirds of our treated patient were controlled. This is in line with some previous studies [32,33,36].

We found an independent correlation between age and hypertension presence, awareness, treatment, and control. Our finding revealed that the 51–70 years old participants were more likely to be hypertensive (adjusted adds ratio = 2.1), more likely to be aware of their diagnosis of hypertension (adjusted adds ratio = 1.8), more likely to be treated (adjusted adds ratio = 1.2), but less likely to be controlled (adjusted adds ratio = 0.6) than 35–50 years old participants. Hypertension increases with the age increasing was a well-known fact and these findings are compatible with all previously published studies [3,31,32,36].

Our results demonstrated that those from urban areas were more likely to be hypertensive (adjusted adds ratio = 1.4) but less likely to be aware (adjusted adds ratio = 0.7) and treated (adjusted adds ratio = 0.6) than those living in rural areas. However, there was no association between residence and hypertension control in the present study. National study in Iran and WHO reports have also revealed that hypertension is more prevalent in urban areas [16,38]. In contrast with our findings, some studies report that rural residency were associated with a greater likelihood of unawareness and uncontrolled of hypertension [39,40]. It is important to note that based on the Health Department program in health networks of Iran, people living in rural areas are actively screened for non-communicable disease, while in cities they are screened passive and that’s may be the reason of more hypertension awareness and treatment in rural areas [41].

Our results revealed that participants with more education than a diploma were less likely to be hypertensive (adjusted adds ratio = 0.8) and were more likely to be aware (adjusted adds ratio = 1.4) and controlled (adjusted adds ratio = 1.4) when compared with high school or less education level. This is consistent with results reported by other studies [32,36].

We found no independent association between sex and risk of hypertension, rate of treatment and control. However, our finding revealed that females were more likely to be aware of their diagnosis of hypertension (adjusted adds ratio = 1.3), than males. Studies reports regarding the correlation of sex with the risk of hypertension are quite controversial [36,42]. Higher levels of awareness in female may be due to their more frequent contact with health care professionals and more attention to their health status [43].

Our results demonstrated that overweight and obese participant were more likely to be hypertensive (adjusted adds ratio = 1.2 and 1.4, respectively), more likely to be aware (adjusted adds ratio = 1.4 and 1.8, respectively) and less likely to be controlled (adjusted adds ratio = 0.6 and 0.4, respectively) than normal weights. Most studies report significant association of overweight, obese with higher risk of hypertension and poor control [44]. However, we found no independent association between abdominal obesity and risk of hypertension, rate of treatment and control. Patient with abdominal obesity had only been more likely to be aware (adjusted adds ratio = 1.5) of their diagnosis of hypertension in our study population. Higher levels of awareness in overweight and obese people may be due to the closer attention of the health system to these groups [31].

Other finding of the current study was an independent correlation between physical activity and hypertension prevalence, awareness and control. Our results revealed that participants with higher physical activity were less likely to be hypertensive (adjusted adds ratio = 0.6), were more likely to be controlled (adjusted adds ratio = 1.6) but were less likely to be aware of the disease (adjusted adds ratio=0.6). Moderate regular physical activity can improve control of blood pressure in 75% of hypertensive patients [45]. The underlying mechanisms are probably reduction in plasma renin and norepinephrine, increased metabolic rate and fat metabolism and reduction in vascular resistance [46,47].

We found no independent association of alcohol use and smoking with risk of hypertension and rate of awareness, treatment and control. It was in contrast with the findings of some studies that showed an independent and significant effect of smoking on hypertension [48,49]. The finding of no association in the current study is maybe due to reverse causation, due to the fact that patient with hypertension are more likely to be advised by their health professions to smoking cessation [50].

The results of current study demonstrate that diabetic patient and patients suffering from CVD were more likely to be hypertensive (adjusted adds ratio = 2.1 and 3.1, respectively), more likely to be aware (adjusted adds ratio = 1.6 and 2, respectively) and treated (adjusted adds ratio = 1.6 and 1.8, respectively), but there was no association of diabetes and CVD with hypertension control. Evidence on the association of diabetes and CVD with hypertension is consistent [51,52]. Higher levels of hypertension awareness and treatment in diabetes and CVD patients can be justified with more frequent refer to health care professionals [36].

We found an independent association between psychiatric disorders and hypertension presence (adjusted adds ratio = 1.5) and awareness (adjusted adds ratio = 1.5). other studies showed that hypertensive patients were more likely to suffer from psychiatric disorders [53,54]. On the other hand, patients who experience stress, depression are at an increased risk of hypertension [55].

Our results demonstrated that those having a family history of hypertension was more likely to be hypertensive (adjusted adds ratio = 1.8), more likely to be aware (adjusted adds ratio = 1.8) and controlled (adjusted adds ratio = 1.8), in line with previous studies [56,57]. This confirms the hereditary pattern of hypertension [58]. Moreover, higher levels of hypertension awareness and control in family history positive patients may be due to frequent contact with health care professionals and more attention to their health status [59].

We found no independent association of serum lipid profile with risk of hypertension and rate of awareness, treatment and control. However, our results revealed a strong correlation of serum creatinine and hypertensive (adjusted adds ratio = 5.1) in line with previous studies [60,61].

The strengths of this study are the large sample size, the population-based data and also the wide collection of information on potentially correlated factors. However, Cross-sectional design was the limitation of the current study, which did not permit us to determine the order of events. Also, we cannot completely exclude residual confounders, despite our detailed adjustment for confounding because our study was not randomized.

### Recommendation for Future Research

Future behavior analysis studies based on the Health Belief Model are recommended to identify barriers of screening and treatment seeking behaviors in general population especially in urban areas and among males, younger ages, and less educated.

## Conclusion

Hypertension is a highly prevalent in the northern Iranian population. About half of affected persons are unaware of their disease and untreated. Modifying risk factors (such as weight lose and increase physical activity), increasing hypertension awareness (by screening) and accessibility to health services is essential for primary and secondary prevention of high blood pressure in this population, especially in urban areas and among males, younger ages, and less educated.
